# The TRPA1 Channel Amplifies the Oxidative Stress Signal in Melanoma

**DOI:** 10.3390/cells10113131

**Published:** 2021-11-11

**Authors:** Francesco De Logu, Daniel Souza Monteiro de Araujo, Filippo Ugolini, Luigi Francesco Iannone, Margherita Vannucchi, Francesca Portelli, Lorenzo Landini, Mustafa Titiz, Vincenzo De Giorgi, Pierangelo Geppetti, Daniela Massi, Romina Nassini

**Affiliations:** 1 Department of Health Sciences, Clinical Pharmacology and Oncology Section, University of Florence, 50139 Florence, Italy; francesco.delogu@unifi.it (F.D.L.); daniel.souzamonteirodearaujo@unifi.it (D.S.M.d.A.); l.landini@unifi.it (L.L.); mustafa.titiz@unifi.it (M.T.); geppetti@unifi.it (P.G.); 2 Advanced Bioimaging Research Laboratory (ABiR), University of Florence, 50139 Florence, Italy; filippo.ugolini@unifi.it (F.U.); daniela.massi@unifi.it (D.M.); 3 Research Unit ChrOnic PAin Laboratory (CoPAL), University of Florence, 50139 Florence, Italy; 4 Department of Health Sciences, Section of Pathological Anatomy, University of Florence, 50139 Florence, Italy; margherita.van@gmail.com (M.V.); fraportelli@gmail.com (F.P.); 5 Headache Center and Clinical Pharmacology, Careggi University Hospital, 50139 Florence, Italy; iannone.luigifrancesco@gmail.com; 6 Division of Dermatology, Azienda Sanitaria Firenze, 50139 Florence, Italy; vincenzo.degiorgi@unifi.it

**Keywords:** oxidative stress, melanoma, macrophages, TRPA1, image analysis

## Abstract

Macrophages (MΦs) and reactive oxygen species (ROS) are implicated in carcinogenesis. The oxidative stress sensor, transient receptor potential ankyrin 1 (TRPA1), activated by ROS, appears to contribute to lung and breast cancer progression. Although TRPA1 expression has been reported in melanoma cell lines, and oxidative stress has been associated with melanocytic transformation, their role in melanoma remains poorly known. Here, we localized MΦs, the final end-product of oxidative stress, 4-hydroxynonenal (4-HNE), and TRPA1 in tissue samples of human common dermal melanocytic nevi, dysplastic nevi, and thin (pT1) and thick (pT4) cutaneous melanomas. The number (amount) of intratumoral and peritumoral M2 MΦs and 4-HNE staining progressively increased with tumor severity, while TRPA1 expression was similar in all samples. Hydrogen peroxide (H_2_O_2_) evoked a TRPA1-dependent calcium response in two distinct melanoma cell lines (SK-MEL-28 and WM266-4). Furthermore, H_2_O_2_ induced a TRPA1-dependent H_2_O_2_ release that was prevented by the TRPA1 antagonist, A967079, or *Trpa1* gene silencing (siRNA). ROS release from infiltrating M2 MΦs may target TRPA1-expressing melanoma cells to amplify the oxidative stress signal that affects tumor cell survival and proliferation.

## 1. Introduction

Cutaneous melanoma is the most common and aggressive subtype of melanoma, arising from malignant transformation of epidermal melanocytes [1]. Macrophages (MΦs) and, particularly, tumor associated MΦs (TAMs) are implicated in all stages of melanogenesis, contributing to cancer progression and metastasis. MΦ increase in the tumor microenvironment (TME) negatively affects the prognosis of the patient with malignant melanoma [2,3,4,5]. TAMs are the major stromal component actively recruited into tumors, where they provide a favorable microenvironment for angiogenesis and cancer cell survival that accelerates tumor progression and metastasis [6,7,8,9]. TAMs can be switched into two distinct phenotypes, namely M1 and M2 [10,11], by labeling with an anti-CD68 (M1-like MΦ marker) and an anti-CD163 (M2-like MΦ marker) antibody [12]. The number of CD68+ve TAMs increases with increasing melanoma invasion and ulceration [13,14,15]. A dense intratumoral infiltration of both CD68+ve and CD163+ve TAMs has been reported in deeply-invasive malignant and metastatic melanomas compared to non-metastatic melanomas [16,17], and in malignant compared to benign melanocytic lesions [18]. MΦ total number is correlated with Breslow thickness, tumor stage, and poor prognosis [13,14,15,16,18].

One major MΦ activity is the generation of reactive oxygen species (ROS) [19], which mediates a variety of either protective (promotion of oxidative stress-induced tumor cell death) or detrimental (tumor cell survival and growth) functions in cancer [20,21]. ROS biotransformation results in a series of molecules, including the reactive carbonylic species (RCS) 4-hydroxynonenal (4-HNE), the end-products of lipid peroxidation of ω-6 polyunsaturated fatty acids [22]. Lipid peroxidation end-products are referred to as “second toxic messengers of free radicals” [23] and have been studied for distinct roles in chemotaxis, autophagy, pain sensation, signal transduction, gene expression, cell proliferation, and death [3,24,25,26,27,28].

Mitochondrial macromolecules derived from 4-HNE are involved in the initiation and progression of cancer [29]. Using monoclonal antibodies specific for 4-HNE protein adducts and biochemical assays, differences in 4-HNE levels between normal and tumoral tissues have been shown in kidney [30], brain [31], colorectal [32], and thyroid [33] cancer. Malignant melanocyte transformation has been associated with oxidative stress, as antioxidant defenses are compromised in pathological conditions [34]. Furthermore, elevated levels of 4-HNE have been reported in a series of five dysplastic nevi and 35 cases of cutaneous malignant melanomas, as compared to five cases of benign nevi [35].

Transient receptor potential ankyrin 1 (TRPA1) belongs to a subgroup of transient receptor potential (TRP) channels of the larger TRP superfamily, which includes vanilloid 1 (TRPV1), TRPV2, TRPV3, TRPV4, and melastatin 8 (TRPM8). TRPA1 possesses the highest oxidation sensitivity [36,37] and behaves as a sensor and amplifier of oxidative stress [38,39]. In addition to its prominent expression and role in somatosensory neurons, TRPA1 has been found in non-neuronal cells [38,40,41,42], including skin cells such as keratinocytes and melanocytes [43,44,45,46]. Recent studies have reported that TRPM8 [47] and TRPA1 [48] exhibit abnormal expression in melanoma cell lines, and functional TRPV1, TRPM8, and TRPA1 have been found in malignant uveal melanoma tissues and cell lines [49]. However, the implication of TRPA1 in the signaling pathway, which from infiltrating MΦs results in increased oxidative stress and its amplification in melanoma, is poorly known.

Here, in a series of common dermal melanocytic nevi (dermal nevi), dysplastic nevi, and thin (pT1) and thick (pT4) malignant melanomas, we simultaneously explored the presence of M1 and M2 TAM subtypes, the level of the oxidative stress end-product 4-HNE, and the expression of the TRPA1 channel. In addition, we studied the expression of TRPA1 in two different melanoma cell lines and its ability to sense and amplify the oxidative stress signal. Results indicate that increased peritumoral and intratumoral M2 TAM are positively associated with 4-HNE levels, and that TRPA1 stimulated by ROS amplifies the oxidative stress signal in cultured melanoma cells.

## 2. Materials and Methods

### 2.1. Tissue Collection

The study cohort included formalin-fixed paraffin-embedded (FFPE) tissues from ten commonly acquired (dermal) melanocytic nevi, nine dysplastic nevi and 28 invasive primary cutaneous melanomas. FFPE samples were retrospectively retrieved from the archive of the Division of Pathology, Department of Health Sciences, University of Florence, Italy. Consecutive cases of nevi, pT1, and pT4 primary cutaneous melanoma, histopathologically diagnosed between 2002 and 2018 at the Section of Anatomic Pathology, Department of Health Sciences, University of Florence, with accessible medical records and sufficient FFPE tumor specimens, were included in the study. In addition, FFPE samples were divided according to TNM classification into two groups according to Breslow thickness (mm): pT1 ≤ 1 mm and pT4 > 4 mm; the specimens showing lentiginous proliferation of melanocytes at the dermoepidermal junction, extending at least three rete ridges beyond lateral margins of the dermal component, were considered dysplastic nevi.

Patients’ data, including age, sex, and anatomic site, were collected. Among patients, 17 were females and 30 were males. The median age of patients with dermal and dysplastic nevi was 35.16 years (range 8–58 years). Dermal nevi site distribution was trunk (*n* = 6) or head & neck (*n* = 4). Dysplastic nevi were all located in the trunk (*n* = 9). The median age of patients with cutaneous melanoma was 56.57 years (range 18–88 years). Tumor site distribution was listed as: extremities (*n* = 10), trunk (*n* = 11), head & neck (*n* = 5), and missing (*n* = 2). They included 17 superficial spreading melanomas, nine nodular melanomas, and two unclassifiable melanomas. Pathological staging (pT) classification was as follows: pT1 (*n* = 13) and pT4 (*n* = 15).

### 2.2. Immunohistochemistry

Immunohistochemistry was performed on representative FFPE whole tumor sections, 3-μm thick, of dermal and dysplastic nevi and invasive primary cutaneous melanoma. For CD68 and CD163 staining, sample processing was performed with automated immunostainer (Ventana Discovery ULTRA, Ventana Medical Systems, Oro Valley, AZ, USA). Sections were deparaffinized in EZ prep (#950-102; Ventana) at 72 °C, and antigen retrieval was achieved by incubation with cell-conditioning solution 1 (#950-124; Ventana, Oro Valley, AZ, USA), a tris ethylenediaminetetraacetic acid-based buffer (pH 8.2). Sections were then incubated with the following primary antibodies: anti-CD68 primary antibody (#DS-0100-B, clone PG-M1, mouse monoclonal, ready to use, Diagnostic BioSystem, Pleasanton, CA, USA) and anti-CD163 (#760-4437, clone MRQ-26, mouse monoclonal, ready to use, Cell Marque, Rocklin, CA, USA). The signal was developed with the UltraMap Red anti-Mouse o Detection Kit (Ventana Medical Systems, Oro Valley, AZ, USA). Sections were counterstained with hematoxylin (#760-2021; Ventana, Oro Valley, AZ, USA). For TRPA1 staining, sample processing was performed manually as follows: antigen retrieval was routinely performed by immersing the slides in a thermostat bath containing 10 mM Citrate Buffer (pH 6.0) for 20 min at 99 °C followed by cooling for 20 min at room temperature. Endogenous peroxidase activity was blocked with a blocking solution (Bloxall, ready to use, Vector, Burlingame, CA, USA) for 10 min. Sections were incubated with anti-TRPA1 antibody (#ACC-037, rabbit polyclonal, 1:100, Alomone, Israel) for 1 h at room temperature. The signal was developed with Vectastain Universal ABC-AP KIT (#AK5200, Vector, Burlingame, CA, USA). Nuclei were counterstained with Mayer’s hematoxylin.

### 2.3. Immunofluorescence

After antigen retrieval (ethylenediaminetetraacetic acid, EDTA, solution pH 9.0) for 20 min at 99 °C, FFPE tissue sections were incubated with an anti-4-HNE (#ab48506, clone (HNEJ-2), mouse monoclonal, 1:25, Abcam, Cambridge, UK) diluted in antibody diluent (Roche Diagnostics, Switzerland) for 1 h at room temperature. Sections were then incubated for 2 h in the dark with fluorescent secondary antibody polyclonal Alexa Fluor 594 (1:600, Invitrogen, Waltham, MA, USA). Sections were coverslipped using a water-based mounting medium with 4′6′-diamidino-2-phenylindole (DAPI, #ab228549, Abcam). Negative controls were performed in tissue samples collected from the analyzed cases, in which the primary antibody was replaced with non-immune Ig serum from the same species as the primary antibody used. The 4-HNE staining was evaluated as the fluorescence intensity, measured by an image processing program (ImageJ 1.32J, https://imagej.nih.gov/ij/, 9 November 2021, National Institutes of Health, Bethesda, MD, USA).

### 2.4. Image Analysis

For each dermal and dysplastic nevi and invasive primary cutaneous melanoma, a representative histopathological whole slide, stained with hematoxylin, eosin, anti-CD68, anti-CD163, and anti-TRPA1, was digitally scanned at 400× magnification with Aperio AT2 (Leica Biosystems, Milan, Italy) into whole slide digital images (WSI). Each SVS format file was imported into Halo Link^®^ (Indica Labs, Albuquerque, NM, USA) image management system. Detection of immune-stained positive cells was performed using Halo^®^ Multiplex immunohistochemistry (IHC) analysis software version v3.1.1076.308, based on cytonuclear features such as stain, size, and roundness. For CD68+ve and CD163+ve cell counting exclusively in the lesion region, we combined the multiplex IHC module with DenseNet AI (plugin) classifier (Indica Labs, Albuquerque, NM, USA). This method allowed us to count immune-stained positive cells in intratumoral and peritumoral areas, excluding any CD68+ve and CD163+ve cells that were outside the lesion, automatically. TRPA1 analysis was performed using the Halo^®^ Area Quantification analysis software version 2.1.7 (Indica Labs, Albuquerque, NM, USA), based on the staining intensity and distribution of TRPA1 staining.

### 2.5. Cell Lines

HEK293 cells, stably transfected with the cDNA for human TRPA1 (hTRPA1-HEK293), were cultured in Minimum Essential Medium (MEM) containing fetal bovine serum (FBS, 10%), l-glutamine (2 mM), penicillin (100 U/mL), and streptomycin (100 mg/mL) under a humidified atmosphere of 5% CO_2_ at 37 °C. The human malignant melanoma SK-MEL-28 cells (American Type Culture Collection, ATCC, #HTB-72™, Manassas, VA, USA) and the human malignant metastatic melanoma WM 266-4 cells (European Collection of Authenticated Cell Cultures, ECACC/Merck Life Science SRL;#9106123, Kenilworth, NJ, USA) were cultured in Dulbecco’s Modified Medium (DMEM) containing FBS (10%), l-glutamine (2 mM), penicillin (100 U/mL), and streptomycin (100 mg/mL) under a humidified atmosphere of 5% CO_2_ at 37 °C. All cells were used without further authentication.

### 2.6. qRT-PCR

Total RNA was extracted from SK-MEL-28 and WM 266-4 cells using the RNeasy Mini kit (Qiagen SpA, Hilden, Germany), according to the manufacturer’s protocol. RNA concentration and purity were assessed spectrophotometrically by measuring the absorbance at 260 nm and 280 nm. RNA was reverse transcribed with the Qiagen QuantiTect Reverse Transcription Kit (Qiagen SpA, Hilden, Germany), following the manufacturer’s protocol. For mRNA relative quantification, rt-PCR was performed on Rotor Gene^®^ Q (Qiagen SpA, Hilden, Germany). The relative abundance of mRNA transcripts was calculated using the delta CT method and normalized to GAPDH levels. The sets of primers were as follows: GAPDH-Seq1 (GAPDH-sequence1): 5′-ACATCGCTCAGACACCATG -3′, GAPDH-Seq2 (GAPDH-sequence2): 5′-TGTAGTTGAGGTCAATGAAGGG-3′ (NCBI Reference Sequence (Ref Seq): NM_002046); TRPA1-Seq15′-GACATTGCTGAGGTCCAGAA-3′, TRPA1-Seq2:5′-GAAACAAAGTGCAGCTTC-3′ (NCBI Ref Seq: NM_007332).

### 2.7. Calcium Imaging

SK-MEL-28 and WM 266-4 cells were seeded on poly-l-lysine (8.3 μM) coated 35 mm glass coverslips (Thermo Fisher, Invitrogen, Waltham, MA, USA) two days before calcium imaging experiments. Plated cells were loaded with Fura-2AM-ester (5 mM, Merck Life Science SRL, Kenilworth, NJ, USA), added to the buffer solution (37 °C) containing the following (in mM): 2 CaCl_2_, 5.4 KCl, 0.4 MgSO_4_, 135 NaCl, 10 d-glucose, 10 HEPES, and 0.1% bovine serum albumin at pH 7.4. After 40 min, cells were washed and transferred to a chamber on the stage of a fluorescent microscope for recording (Olympus IX 81). Cells were challenged with the selective TRPA1 agonist, allyl isothiocyanate (AITC 50 nM–10 mM), and the oxidative stress bioproduct, hydrogen peroxide (H_2_O_2_ 10 μM–10 mM). The calcium response was monitored in the presence of A967079 (30 μM) or vehicle (0.3% dimethyl sulfoxide, DMSO). Results were expressed as % increase in Ratio_340/380_ (R_340/380_) over baseline normalized to the maximum effect induced by ionomycin (5 μM) added at the end of each experiment (% change in R_340/380_).

### 2.8. siRNA Transfection

The TriFECTa^®^ RNAi kit (Integrated DNA Technologies, IDT, Coralville, IA, USA) was used to silence the Trpa1 gene in SK-MEL-28 and WM 266-4 cell lines. Transfections of siRNA into cells were performed according to each manufacturer’s instructions. Briefly, one day before the transfection, SK-MEL-28 and WM 266-4 were plated to 6-well plates (1 × 10^6^ cells/well) in 2.5 mL of growth medium without antibiotics until 70–80% of confluence. Cells were transfected as follows: siRNA and lipofectamine RNAiMAX (#13778-075, Life Technologies, Waltham, MA, USA) were diluted in Opti-MEM I Reduced Serum Medium (#31985-047, Gibco^®^-Life Technologies, Carlsbad, CA, USA) separately before being mixed by pipetting. The final medium amount per well was 2.5 mL. The siRNA-RNAiMAX mix was added to 6-well cell culture plates. The final concentrations of lipofectamine RNAiMAX and siRNA were 3 μL/mL and 10 nM, respectively. Cells were then incubated for 24 h under a humidified atmosphere of 5% CO_2_ at 37 °C. Knock-down efficiency was detected at 24 h by qRT-PCR comparative quantitation analysis.

### 2.9. Protein Extraction and Western Immunoblot Assay

hTRPA1-HEK293, SK-MEL-28 and WM266-4 cells were plated on 30 mm culture dishes and maintained in 5% CO_2_ and 95% O_2_ (37 °C, until confluence). The cells were homogenized in an RIPA buffer (NaCl (150 mM), Tris-base (50 mM), EGTA (5 mM), Triton X-100 (1%), sodium deoxycholate (0.5%), sodium dodecyl sulfate (0.1%)) containing dithiothreitol (1 mM), and complete protease inhibitor cocktail (Merck Life Science SRL). Lysates were centrifuged at 12,000 rpm at 4 °C for 10 min. Protein concentration in supernatants was determined using a BCA protein assay (Thermo Scientific, Waltham, MA, USA). Samples with equal amounts of proteins (20 μg) were then separated by NuPAGE 4–12% bis-tris gel electrophoresis (Life Technologies), and the proteins were transferred to a nitrocellulose (Bio-Rad). Membranes were incubated with dry milk (5%) in Tris buffer (TBST; Tris (20 mM) at pH 7.5, NaCl (150 mM)) containing Tween 20 (0.1%), for 1 h at room temperature, and incubated with the following primary antibodies: anti-TRPA1 (#ACC-037, rabbit polyclonal, 1:200, Alomone, Israel) or β-actin (#ab6276, mouse monoclonal, 1:5000, Abcam) at 4 °C overnight. Membranes were then probed with goat anti-mouse or donkey anti-rabbit IgG conjugated with horseradish peroxidase (HRPO, 1:10,000, Bethyl Laboratories Inc., Montgomery, TX, USA) for 2 h at room temperature. Finally, membranes were washed three times with TBST, and bound antibodies were detected using chemiluminescence reagents (Pierce™ ECL, Thermo Scientific) and revealed using an imaging system (ChemiDoc, BioRad, Hercules, CA, USA). The density of specific bands was measured using an image processing program (ImageJ 1.32J, National Institutes of Health) and normalized to β-actin. The uncropped scan of the blot is reported in the Supplementary Figure S1.

### 2.10. H_2_O_2_ Assay

H_2_O_2_ was determined by using the Amplex Red assay (#A12222, Thermo Fisher, Invitrogen). SK-MEL-28 and WM 266-4 cells were plated in 96-well black wall clear bottom plates (Corning Life Sciences, Glendale, AZ, USA) (5 × 105 cells/well) and maintained in 5% CO_2_ and 95% O_2_ (24 h, 37 °C). The cultured medium was replaced with Krebs-Ringer phosphate (KRP, composition in mM: 2 CaCl_2_; 5.4 KCl; 0.4 MgSO_4_; 135 NaCl; 10 d-glucose; 10 HEPES (pH 7.4)) added with A967079 (30 μM) or vehicle (0.3% DMSO in KRP) for 20 min at room temperature. SK-MEL-28 and WM 266-4 cells were then stimulated with AITC (30 μM), H_2_O_2_ (200 nM), or their vehicles (0.03% DMSO and KRP, respectively), added with Amplex red (50 μM) and horseradish peroxidase (HRP, 1 U/mL), and maintained for 40 min at room temperature protected from light. Some experiments were performed with SK-MEL-28 and WM 266-4 after *Trpa1* silencing. Signal was detected 60 min after exposure to the stimuli. H_2_O_2_ release was calculated using H_2_O_2_ standards and expressed as nmol/1. To evaluate changes produced by the release of endogenous H_2_O_2_ from melanoma cells following stimulation, baseline level of H_2_O_2_ 200 nmol was measured in the medium in the absence of cells.

### 2.11. Statistical Analysis

Results are expressed as mean ± standard error of the mean (SEM). For multiple comparisons, a one-way analysis of variance (ANOVA) followed by the post-hoc Bonferroni’s test or Dunnett’s test was used. Statistical analyses were performed on raw data using Graph Pad Prism 8 (GraphPad Software Inc., San Diego, CA, USA). EC_50_ values were determined from non-linear regression models using Graph Pad Prism 8 (GraphPad Software Inc.). A Spearman’s rank-order correlation was run to assess the relationship between 4-HNE and CD68+ve or CD163+ve cells, and data was visualized with simple scatterplots (IBM Corp. SPSS Statistics, version 26). *p* values less than 0.05 (*p* < 0.05) were considered significant. Statistical tests used and the sample size for each analysis are listed in the figure legends.

## 3. Results

### 3.1. TAM Quantification in Dermal and Dysplastic Nevi, and Melanoma Tissues

**Figure 1:**
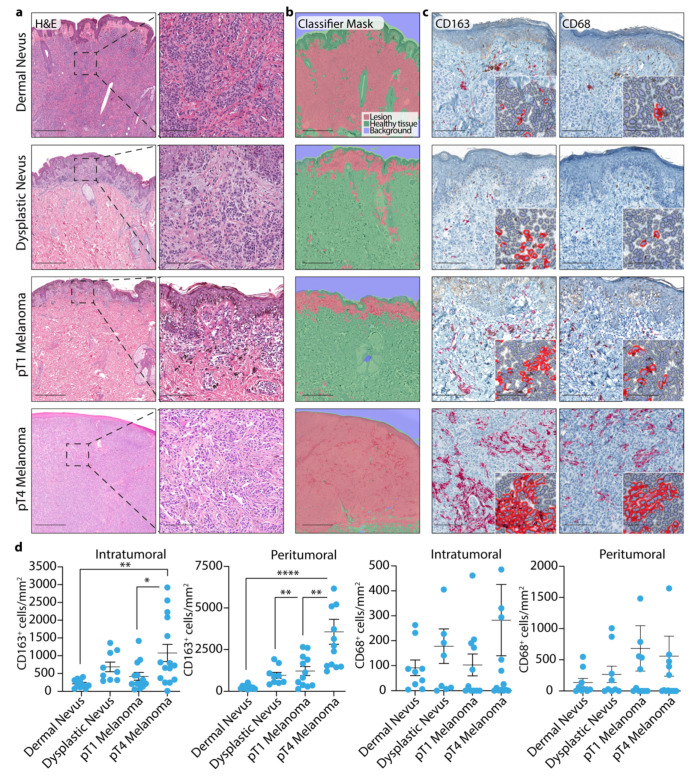
Digital quantification of CD163+ve and CD68+ve cells in dermal and dysplastic nevi and melanoma tissues. (**a**) Representative images of hematoxylin and eosin (H&E) staining in dermal and dysplastic nevus and pT1 and pT4 melanoma tissues. Scale bar represents 500 μm and 100 µm. (**b**) Representative images of Halo artificial intelligence-AI^®^ automatic segmentation of a lesion, healthy tissue and background of dermal and dysplastic nevus and pT1 and pT4 melanoma tissues. Scale bar 500 μm. (**c**) Representative images of intratumoral CD68+ve and CD163+ve cells with digital automatic quantification mask of positive cells (inset) and (**d**) pooled data of intratumoral and peritumoral CD68+ve and CD163+ve cells. Scale bar 500 μm and 50 µm (inset). * *p* < 0.05, ** *p* < 0.01 **** *p* < 0.0001, one-way ANOVA and Bonferroni post hoc test.

We first investigated, by immunohistochemistry, the density of CD163+ve and CD68+ve TAMs, separately, in both the intratumoral and peritumoral compartment. We observed that CD163+ve TAMs were higher in thicker pT4 melanomas than those found in dermal nevi (Figure 1a–d). A significant increase of CD163+ve TAMs was also observed between deeply invasive (pT4) and thin (pT1) melanomas (Figure 1a–d). Although a tendency to higher numbers of CD68+ve TAMs was observed from dermal nevi to pT4 melanomas, no significant difference in the four categories of samples analyzed was found (Figure 1a–d).

### 3.2. 4-HNE Levels in Dermal and Dysplastic Nevi, and Melanoma Tissues and the Correlation with TAMs

**Figure 2:**
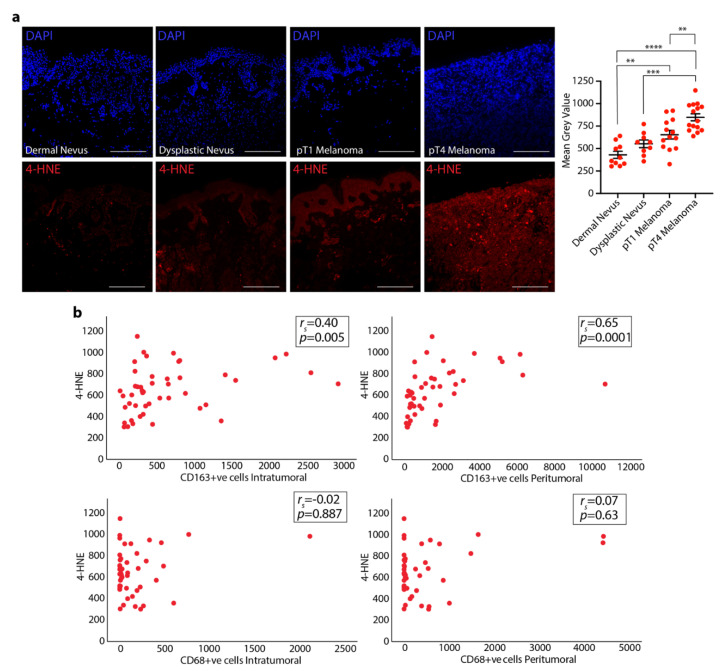
4-HNE expression in dermal and dysplastic nevi and melanoma tissues and its correlation with TAMs. (**a**) Representative images and pooled data of 4-HNE staining in dermal and dysplastic nevus and pT1 and pT4 melanoma tissues. DAPI is the nuclear counterstaining. (**b**) Scatter plot reporting Spearman’s correlation between 4-HNE and intratumoral and peritumoral CD68+ve or CD163+ve cells. Scale bar 250 μm. ** *p* < 0.01, *** *p* < 0.001, **** *p* < 0.0001, one-way ANOVA and Bonferroni post hoc test.

The presence of the oxidative stress byproduct, 4-HNE, was explored in dermal and dysplastic nevi and melanoma tissues. Immunofluorescence analysis revealed that 4-HNE expression was significantly higher in pT1 melanomas compared to dermal nevi and in pT4 melanomas compared to dermal and dysplastic nevi (Figure 2a). In addition, thicker (pT4) melanomas showed an increased 4-HNE staining compared to thinner (pT1) melanomas (Figure 2a).

By running a Spearman’s correlation, we assessed the relationship between 4-HNE and CD68+ve or CD163+ve cells in the four different types of samples. A positive, statistically significant correlation between 4-HNE and CD163+ve cells was found in samples from dermal and dysplastic nevi and pT1 and pT4 melanomas. In particular, there was a moderate positive correlation between 4-HNE and intratumoral CD163+ve cells (*rs* = 0.405, *n* = 47, *p* = 0.005) and a stronger positive correlation between 4-HNE and peritumoral CD163+ve cells (*rs* = 0.650, *n* = 45, *p* < 0.0001). No correlation was found between 4-HNE and either intratumoral CD68+ve cells (*rs* = −0.022, *n* = 45, *p* = 0.887) or peritumoral CD68+ve cells (*rs* = 0.073, *n* = 44, *p* = 0.636) (Figure 2b).

### 3.3. Expression and Function of TRPA1 in Dermal and Dysplastic Nevi, Melanoma Tissues, and Melanoma Cell Lines

**Figure 3:**
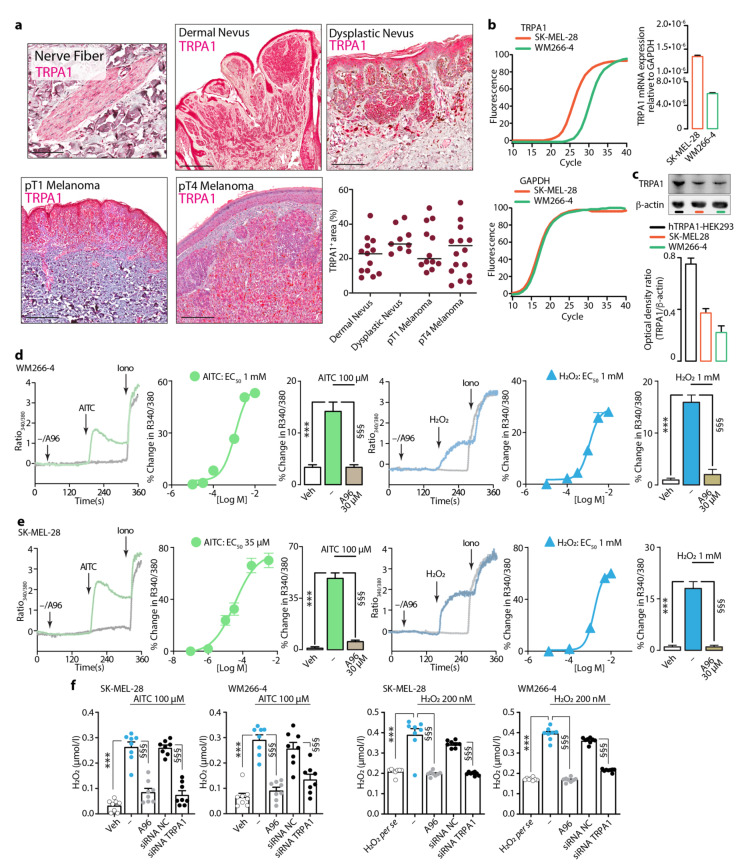
TRPA1 expression and function in dermal and dysplastic nevi and melanoma tissues and melanoma cell lines. (**a**) Representative images of TRPA1 staining in cutaneous nerve fiber (scale bar 50 µm) and representative images and cumulative data of the automatic staining area quantification in dermal and dysplastic nevus, and pT1 and pT4 melanoma tissues (scale bar 500 μm). (**b**) Representative curve and cumulative data of TRPA1 mRNA relative expression in WM266-4 and SK-MEL-28 cell lines. (**c**) Representative image of western immunoblot and TRPA1 protein content in cultured hTRPA1-HEK293, SK-MEL-28 and WM266-4 cell lines. Typical traces, concentration-response curve and pooled data of the calcium response evoked by AITC and H_2_O_2_ in the presence of the TRPA1 antagonist, A967079 (A96) or its vehicle in WM-266-4 (**d**) and SK-MEL-28 (**e**) melanoma cells. (**f**) H_2_O_2_ release from WM-266-4 and SK-MEL-28 melanoma cells induced by AITC or H_2_O_2_ in presence of A96 (30 μM) or its vehicle and after *Trpa1* siRNA silencing or siRNA negative control (NC). Iono represents ionomycin; H_2_O_2_ *per se* represents the value of H_2_O_2_ not in presence of cells. Veh is the vehicle of AITC or H_2_O_2_, hyphen (-) is the vehicle of A96. *** *p* < 0.001, ^§§§^ *p* < 0.001 one-way ANOVA and Bonferroni post hoc test.

The expression of TRPA1, a major chemosensory receptor for ROS [50], was evaluated in tissue samples of dermal and dysplastic nevi and pT1 and pT4 malignant melanomas. In skin adjacent to the lesion, TRPA1 was found in cutaneous nerves, which are used as staining positive control (Figure 3a). Normal melanocytes generally showed faint to moderate TRPA1 staining, although the immunolabelling was occasionally difficult to evaluate, due to the presence of a clear perinuclear halo, resulting from marked cytoplasmic retraction. In dermal and dysplastic nevi, as well as in melanoma cells, TRPA1 immunoreactivity was comparable and mostly confined to the cell cytoplasm and peripheral (plasma or cell membrane) membrane without nuclear pattern (Figure 3a). However, the quantification of TRPA1+ve marked area revealed no significant differences in TRPA1 expression between the analyzed samples (Figure 3a).

Finally, the expression and functional activity of TRPA1 was evaluated in two different melanoma cell lines, SK-MEL-28 (malignant melanoma) and WM266-4 (derived from a metastatic site of a malignant melanoma) cells. TRPA1 mRNA (Figure 3b) and protein (Figure 3c) were found in both cell lines. To verify that the TRPA1 mRNA was translated into a functional protein, we evaluated the calcium response to two channel agonists, allyl isothiocyanate (AITC) and hydrogen peroxide (H_2_O_2_). Both compounds induced a concentration-dependent calcium response that was abolished by the selective TRPA1 channel antagonist, A967079 (Figure 3d,e).

TRPA1 activation by electrophilic and reactive endogenous and exogenous agents results in a calcium-dependent NOX1 stimulation that amplifies the oxidative stress signal [25,38,51]. Here, we asked whether TRPA1 could increase ROS production in the two different melanoma cell lines. Exposure of SK-MEL-28 and WM266-4 cells to either AITC or H_2_O_2_ increased H_2_O_2_ content in the medium, a response that was prevented by the TRPA1 selective antagonist, A967079, and by Trpa1 gene silencing (siRNA) (Figure 3f). Thus, a functional TRPA1 channel expressed by melanoma cells is targeted by ROS to amplify the oxidative stress signal.

## 4. Discussion

While proinflammatory M1 MΦs are mainly involved in the initial phase of cancer development by creating a mutagenic microenvironment [52], in more advanced stages of cancer, TAMs often differentiate into anti-inflammatory M2 MΦs, which enhance tumor growth by creating an immunosuppressive TME by producing proangiogenic molecules and proteolytic enzymes to promote angiogenesis and tumor invasion [10]. A higher number of CD68+ve TAMs has been reported with increasing depth of melanoma invasion and ulceration [13,14,15] and in malignant compared to benign melanocytic lesions [18]. Both CD68+ve and CD163+ve TAMs were more abundant in metastatic compared to non-metastatic melanomas [16,17], and in malignant compared to benign melanocytic lesions [18], a finding which has been associated with tumor progression and metastasis aas well as poor clinical outcome. A massive MΦ infiltration has been linked to angiogenesis in cutaneous melanoma, as the numbers of MΦs and neovascularization and vascular endothelial growth factor-A increased significantly with augmented tumor depth [13,14]. A dense CD163+ve MΦ infiltration in melanoma stromal tissue and CD68+ve MΦ infiltration at the invasive front were associated with poor overall survival [16]. Moreover, high MΦ counts correlated with markers of aggressive disease, such as Breslow thickness, ulceration, mitotic rate [15], and tumor recurrence [18].

Here, we confirmed that higher numbers of CD163+ve TAMs are associated with a more invasive melanoma phenotype, as indicated by Breslow thickness. The higher density of CD163+ve TAMs in thicker pT4 melanomas, in comparison with thinner pT1 melanomas and dermal nevi, might suggest an association between immunosuppression and cancer progression. However, different from a previous study [18], we only found a tendency to an increased density of CD68+ve TAMs from dermal and dysplastic nevi to pT1 and pT4 melanomas. Failure to reach statistical significance may, in part, depend on the limited sample size and intrinsic variability of study materials. MΦs are among the major sources of oxidative stress [19], which may exert antioncogenic roles [21,53]. However, opposing evidence proposes that ROS exert selective pressure on the tumor cells and orchestrate cellular signaling networks that may aggravate tumor cell proliferation, malignancy, and drug resistance [54,55,56]. Compared to other types of tumors, melanoma is relatively unique, due to its extraordinarily high levels of oxidative stress in TME [55]. A previous comparative analysis of 4-HNE expression revealed a low level in common nevi that significantly increased in dysplastic nevi and malignant melanomas, while melanoma metastases lost the 4-HNE content [35].

We found progressively increasing levels of 4-HNE in association with severity of disease, with more intense 4-HNE staining in deeply invasive pT4 than in thin pT1 melanomas. Although these results suggest an intimate relationship between 4-HNE and the tumorigenesis process, they cannot determine whether the biomarker exerts a proactive role or remains a functionless end-product of tumor-associated oxidative stress. In malignant and advanced tumors, TAMs are biased toward the M2 phenotype, which favors tumor malignancy [11,57,58], and the increased oxidative stress is intimately linked to tumor progression. Thus, the most parsimonious explanation is that increased 4-HNE levels result from different components. Initially, the oxidative stress burden is generated mainly by invading peritumoral and intratumoral MΦs, with a possible higher contribution of CD163+ve cells. In a second phase, TRPA1, in melanoma cells targeted by ROS from MΦs, amplifies the oxidative stress in TME.

Recently, altered expression of a subset of TRP channels has been described throughout various cancer types, where their presence may confer selective growth and survival advantages to tumor cells, thus promoting cancer development and progression [59]. Some TRPs may regulate cellular functions of human melanoma. TRPM2, TRPM8, TRPV1, and TRPV2 have been identified in melanoma cell lines, where their expression and function confer susceptibility to apoptosis and necrosis [47,60,61]. Functional TRPV1, TRPM8, and TRPA1 were also expressed in malignant human uveal melanoma tissues and cell lines [49].

Limitations of the present study are: (1) the TRPA1 antibody was not extensively used in human tumor samples and (2) in vitro data were obtained in melanoma cell lines and not in primary cultures of melanoma cells. Nevertheless, given the fundamental role of ROS in a wide range of cellular responses, including tumor cell proliferation, migration, and survival [54,55], and a prominent role of TRPA1 as a sensor and amplifier of oxidative stress [25,37,38,51], we explored the role of TRPA1 in regulating oxidative stress production in melanoma cells. The expression of TRPA1 in two different melanoma cell lines was associated with their ability to evoke a calcium response by channel agonists AITC and H_2_O_2_. Importantly, H_2_O_2_ elicited a TRPA1-dependent H_2_O_2_ release, as it was attenuated by pharmacological antagonism or gene silencing of the channel. These findings suggest that, in melanoma, as reported under circumstances of inflammation or tissue injury [38,51], TRPA1 behaves as an oxidative stress sensor and amplifier.

**Figure 4:**
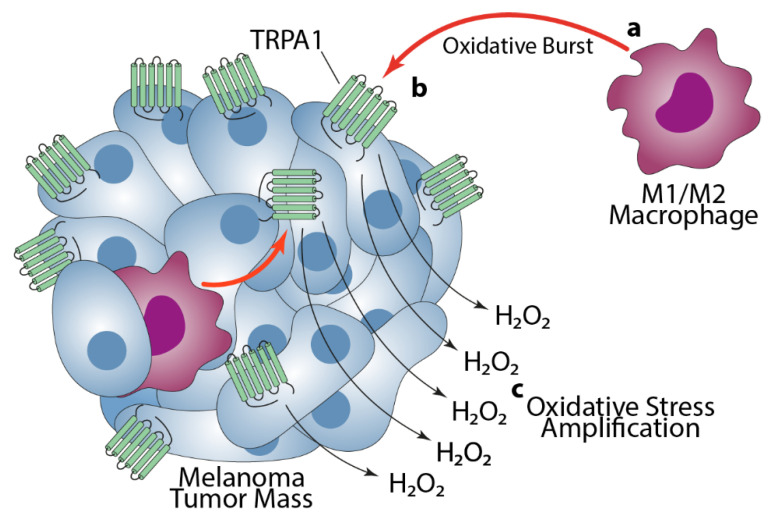
(**a**) Oxidative burst generated by invading peritumoral and intratumoral, M1 and M2 macrophages (**b**) targets TRPA1 on melanoma cells which (**c**) amplifies the oxidative stress (H_2_O_2_) signal to favor tumor progression.

TRPA1 activation is associated with a non-canonical oxidative-stress defense, as well as a canonical ROS-neutralizing mechanism, thus upregulating anti-apoptotic pathways that favor cancer progression [36]. However, there is also evidence that highlights the pro-oncogenic role of oxidative stress in melanoma [62]. Solar ultraviolet radiation, a major risk factor for melanoma [63], elicits melanin formation via TRPA1 in melanocytes [46]. TRPA1, which exhibits the highest sensitivity to oxidants due to the presence of hyperreactive cysteines in cytoplasmic domains [36,37], previously [48] and in this study, has been found functionally expressed in melanoma cells. Our finding that H_2_O_2_ elicits a TRPA1-dependent calcium response in melanoma cells extends to melanoma the hypothesis [36] that TRPA1 promotes antiapoptotic pro-oncogenic programs. However, our additional result that channel activation by H_2_O_2_ in melanoma cell lines amplifies the oxidative stress signal underlines the need of further studies to fully understand the complex and sometimes opposing relationship between MΦs, oxidative stress, and TRPA1 in the initiation and progression of some tumors, including melanoma (Figure 4).

## Data Availability

Data presented in this study are available on request from the corresponding author.

## Supplementary Materials

The following are available online at https://www.mdpi.com/article/10.3390/cells10113131/s1, Figure S1: The uncropped scan of the blot.

## References

[1] Hayat M.J., Howlader N., Reichman M.E., Edwards B.K. (2007). Cancer statistics, trends, and multiple primary cancer analyses from the Surveillance, Epidemiology, and End Results (SEER) Program. Oncologist.

[2] Chen Y., Song Y., Du W., Gong L., Chang H., Zou Z. (2019). Tumor-associated macrophages: An accomplice in solid tumor progression. J. Biomed. Sci..

[3] Dodson M., Wani W.Y., Redmann M., Benavides G.A., Johnson M.S., Ouyang X., Cofield S.S., Mitra K., Darley-Usmar V., Zhang J. (2017). Regulation of autophagy, mitochondrial dynamics, and cellular bioenergetics by 4-hydroxynonenal in primary neurons. Autophagy.

[4] Yan K., Wang Y., Lu Y., Yan Z. (2021). Coexpressed Genes That Promote the Infiltration of M2 Macrophages in Melanoma Can Evaluate the Prognosis and Immunotherapy Outcome. J. Immunol. Res..

[5] De Logu F., Galli F., Nassini R., Ugolini F., Simi S., Cossa M., Miracco C., Gianatti A., De Giorgi V., Rulli E. (2021). Digital Immunophenotyping Predicts Disease Free and Overall Survival in Early Stage Melanoma Patients. Cells.

[6] Pollard J.W. (2004). Tumour-educated macrophages promote tumour progression and metastasis. Nat. Rev. Cancer.

[7] Mantovani A., Sica A. (2010). Macrophages, innate immunity and cancer: Balance, tolerance, and diversity. Curr. Opin. Immunol..

[8] Van Ginderachter J.A., Movahedi K., Hassanzadeh Ghassabeh G., Meerschaut S., Beschin A., Raes G., De Baetselier P. (2006). Classical and alternative activation of mononuclear phagocytes: Picking the best of both worlds for tumor promotion. Immunobiology.

[9] Movahedi K., Laoui D., Gysemans C., Baeten M., Stangé G., Van den Bossche J., Mack M., Pipeleers D., In’t Veld P., De Baetselier P. (2010). Different tumor microenvironments contain functionally distinct subsets of macrophages derived from Ly6C(high) monocytes. Cancer Res..

[10] Chanmee T., Ontong P., Konno K., Itano N. (2014). Tumor-associated macrophages as major players in the tumor microenvironment. Cancers.

[11] Biswas S.K., Mantovani A. (2010). Macrophage plasticity and interaction with lymphocyte subsets: Cancer as a paradigm. Nat. Immunol..

[12] Aras S., Zaidi M.R. (2017). TAMeless traitors: Macrophages in cancer progression and metastasis. Br. J. Cancer.

[13] Torisu H., Ono M., Kiryu H., Furue M., Ohmoto Y., Nakayama J., Nishioka Y., Sone S., Kuwano M. (2000). Macrophage infiltration correlates with tumor stage and angiogenesis in human malignant melanoma: Possible involvement of TNFalpha and IL-1alpha. Int. J. Cancer.

[14] Varney M.L., Johansson S.L., Singh R.K. (2005). Tumour-associated macrophage infiltration, neovascularization and aggressiveness in malignant melanoma: Role of monocyte chemotactic protein-1 and vascular endothelial growth factor-A. Melanoma Res..

[15] Storr S.J., Safuan S., Mitra A., Elliott F., Walker C., Vasko M.J., Ho B., Cook M., Mohammed R.A., Patel P.M. (2012). Objective assessment of blood and lymphatic vessel invasion and association with macrophage infiltration in cutaneous melanoma. Mod. Pathol..

[16] Jensen T.O., Schmidt H., Moller H.J., Hoyer M., Maniecki M.B., Sjoegren P., Christensen I.J., Steiniche T. (2009). Macrophage markers in serum and tumor have prognostic impact in American Joint Committee on Cancer stage I/II melanoma. J. Clin. Oncol..

[17] Emri E., Egervari K., Varvolgyi T., Rozsa D., Miko E., Dezso B., Veres I., Mehes G., Emri G., Remenyik E. (2013). Correlation among metallothionein expression, intratumoural macrophage infiltration and the risk of metastasis in human cutaneous malignant melanoma. J. Eur. Acad. Dermatol. Venereol..

[18] Salmi S., Siiskonen H., Sironen R., Tyynela-Korhonen K., Hirschovits-Gerz B., Valkonen M., Auvinen P., Pasonen-Seppanen S. (2019). The number and localization of CD68+ and CD163+ macrophages in different stages of cutaneous melanoma. Melanoma Res..

[19] Forman H.J., Torres M. (2001). Redox signaling in macrophages. Mol. Aspects Med..

[20] Kamata T. (2009). Roles of Nox1 and other Nox isoforms in cancer development. Cancer Sci..

[21] Xu Q., Choksi S., Qu J., Jang J., Choe M., Banfi B., Engelhardt J.F., Liu Z.G. (2016). NADPH Oxidases Are Essential for Macrophage Differentiation. J. Biol. Chem..

[22] Esterbauer H., Schaur R.J., Zollner H. (1991). Chemistry and biochemistry of 4-hydroxynonenal, malonaldehyde and related aldehydes. Free Radic. Biol. Med..

[23] Jaganjac M., Tirosh O., Cohen G., Sasson S., Zarkovic N. (2013). Reactive aldehydes--second messengers of free radicals in diabetes mellitus. Free Radic. Res..

[24] Curzio M., Esterbauer H., Di Mauro C., Cecchini G., Dianzani M.U. (1986). Chemotactic activity of the lipid peroxidation product 4-hydroxynonenal and homologous hydroxyalkenals. Biol. Chem. Hoppe. Seyler.

[25] De Logu F., De Pra S.D., de David Antoniazzi C.T., Kudsi S.Q., Ferro P.R., Landini L., Rigo F.K., de Bem Silveira G., Silveira P.C.L., Oliveira S.M. (2020). Macrophages and Schwann cell TRPA1 mediate chronic allodynia in a mouse model of complex regional pain syndrome type I. Brain Behav. Immun..

[26] Marone I.M., De Logu F., Nassini R., De Carvalho Goncalves M., Benemei S., Ferreira J., Jain P., Li Puma S., Bunnett N.W., Geppetti P. (2018). TRPA1/NOX in the soma of trigeminal ganglion neurons mediates migraine-related pain of glyceryl trinitrate in mice. Brain.

[27] Fazio V.M., Barrera G., Martinotti S., Farace M.G., Giglioni B., Frati L., Manzari V., Dianzani M.U. (1992). 4-Hydroxynonenal, a product of cellular lipid peroxidation, which modulates c-myc and globin gene expression in K562 erythroleukemic cells. Cancer Res..

[28] Hammer A., Ferro M., Tillian H.M., Tatzber F., Zollner H., Schauenstein E., Schaur R.J. (1997). Effect of oxidative stress by iron on 4-hydroxynonenal formation and proliferative activity in hepatomas of different degrees of differentiation. Free Radic. Biol. Med..

[29] Ohnishi S., Ma N., Thanan R., Pinlaor S., Hammam O., Murata M., Kawanishi S. (2013). DNA damage in inflammation-related carcinogenesis and cancer stem cells. Oxid. Med. Cell Longev..

[30] Oberley T.D., Toyokuni S., Szweda L.I. (1999). Localization of hydroxynonenal protein adducts in normal human kidney and selected human kidney cancers. Free Radic. Biol. Med..

[31] Zarkovic K., Juric G., Waeg G., Kolenc D., Zarkovic N. (2005). Immunohistochemical appearance of HNE-protein conjugates in human astrocytomas. Biofactors.

[32] Skrzydlewska E., Stankiewicz A., Sulkowska M., Sulkowski S., Kasacka I. (2001). Antioxidant status and lipid peroxidation in colorectal cancer. J. Toxicol. Environ. Health A.

[33] Young O., Crotty T., O’Connell R., O’Sullivan J., Curran A.J. (2010). Levels of oxidative damage and lipid peroxidation in thyroid neoplasia. Head Neck.

[34] Denat L., Kadekaro A.L., Marrot L., Leachman S.A., Abdel-Malek Z.A. (2014). Melanocytes as instigators and victims of oxidative stress. J. Investig. Dermatol..

[35] Blendea A., Serban I.L., Brănişteanu D.C., Brănişteanu D.E. (2017). Evaluation of Immunostaining for 4-Hydroxy-2-Nonenal Receptors in Cutaneous Malignant Melanoma Immunohistochemical Study of 55 Cases. J. Mol. Biomark. Diagn..

[36] Takahashi N., Chen H.Y., Harris I.S., Stover D.G., Selfors L.M., Bronson R.T., Deraedt T., Cichowski K., Welm A.L., Mori Y. (2018). Cancer Cells Co-opt the Neuronal Redox-Sensing Channel TRPA1 to Promote Oxidative-Stress Tolerance. Cancer Cell.

[37] Takahashi N., Mori Y. (2011). TRP Channels as Sensors and Signal Integrators of Redox Status Changes. Front. Pharmacol..

[38] De Logu F., Nassini R., Materazzi S., Carvalho Goncalves M., Nosi D., Rossi Degl’Innocenti D., Marone I.M., Ferreira J., Li Puma S., Benemei S. (2017). Schwann cell TRPA1 mediates neuroinflammation that sustains macrophage-dependent neuropathic pain in mice. Nat. Commun..

[39] Souza Monteiro de Araujo D., De Logu F., Adembri C., Rizzo S., Janal M.N., Landini L., Magi A., Mattei G., Cini N., Pandolfo P. (2020). TRPA1 mediates damage of the retina induced by ischemia and reperfusion in mice. Cell Death Dis..

[40] Earley S., Brayden J.E. (2015). Transient receptor potential channels in the vasculature. Physiol. Rev..

[41] Nassini R., Pedretti P., Moretto N., Fusi C., Carnini C., Facchinetti F., Viscomi A.R., Pisano A.R., Stokesberry S., Brunmark C. (2012). Transient receptor potential ankyrin 1 channel localized to non-neuronal airway cells promotes non-neurogenic inflammation. PLoS ONE.

[42] Nozawa K., Kawabata-Shoda E., Doihara H., Kojima R., Okada H., Mochizuki S., Sano Y., Inamura K., Matsushime H., Koizumi T. (2009). TRPA1 regulates gastrointestinal motility through serotonin release from enterochromaffin cells. Proc. Natl. Acad. Sci. USA.

[43] Atoyan R., Shander D., Botchkareva N.V. (2009). Non-neuronal expression of transient receptor potential type A1 (TRPA1) in human skin. J. Investig. Dermatol..

[44] Radtke C., Sinis N., Sauter M., Jahn S., Kraushaar U., Guenther E., Rodemann H.P., Rennekampff H.O. (2011). TRPV channel expression in human skin and possible role in thermally induced cell death. J. Burn Care Res..

[45] Sokabe T., Tominaga M. (2010). The TRPV4 cation channel: A molecule linking skin temperature and barrier function. Commun. Integr. Biol..

[46] Bellono N.W., Kammel L.G., Zimmerman A.L., Oancea E. (2013). UV light phototransduction activates transient receptor potential A1 ion channels in human melanocytes. Proc. Natl. Acad. Sci. USA.

[47] Yamamura H., Ugawa S., Ueda T., Morita A., Shimada S. (2008). TRPM8 activation suppresses cellular viability in human melanoma. Am. J. Physiol. Cell. Physiol..

[48] Oehler B., Scholze A., Schaefer M., Hill K. (2012). TRPA1 is functionally expressed in melanoma cells but is not critical for impaired proliferation caused by allyl isothiocyanate or cinnamaldehyde. Naunyn Schmiedebergs Arch. Pharmacol..

[49] Mergler S., Derckx R., Reinach P.S., Garreis F., Bohm A., Schmelzer L., Skosyrski S., Ramesh N., Abdelmessih S., Polat O.K. (2014). Calcium regulation by temperature-sensitive transient receptor potential channels in human uveal melanoma cells. Cell Signal..

[50] Bessac B.F., Sivula M., von Hehn C.A., Escalera J., Cohn L., Jordt S.E. (2008). TRPA1 is a major oxidant sensor in murine airway sensory neurons. J. Clin. Investig..

[51] De Logu F., Marini M., Landini L., Souza Monteiro de Araujo D., Bartalucci N., Trevisan G., Bruno G., Marangoni M., Schmidt B.L., Bunnett N.W. (2021). Peripheral Nerve Resident Macrophages and Schwann Cells Mediate Cancer-Induced Pain. Cancer Res..

[52] Noy R., Pollard J.W. (2014). Tumor-associated macrophages: From mechanisms to therapy. Immunity.

[53] Aggarwal V., Tuli H.S., Varol A., Thakral F., Yerer M.B., Sak K., Varol M., Jain A., Khan M.A., Sethi G. (2019). Role of Reactive Oxygen Species in Cancer Progression: Molecular Mechanisms and Recent Advancements. Biomolecules.

[54] Wittgen H.G., van Kempen L.C. (2007). Reactive oxygen species in melanoma and its therapeutic implications. Melanoma Res..

[55] Fruehauf J.P., Trapp V. (2008). Reactive oxygen species: An Achilles’ heel of melanoma?. Expert Rev. Anticancer Ther..

[56] Storz P. (2005). Reactive oxygen species in tumor progression. Front. Biosci..

[57] Qian B.Z., Pollard J.W. (2010). Macrophage diversity enhances tumor progression and metastasis. Cell.

[58] Rolny C., Mazzone M., Tugues S., Laoui D., Johansson I., Coulon C., Squadrito M.L., Segura I., Li X., Knevels E. (2011). HRG inhibits tumor growth and metastasis by inducing macrophage polarization and vessel normalization through downregulation of PlGF. Cancer Cell.

[59] Park Y.R., Chun J.N., So I., Kim H.J., Baek S., Jeon J.H., Shin S.Y. (2016). Data-driven Analysis of TRP Channels in Cancer: Linking Variation in Gene Expression to Clinical Significance. Cancer Genomics Proteomics.

[60] Orfanelli U., Wenke A.K., Doglioni C., Russo V., Bosserhoff A.K., Lavorgna G. (2008). Identification of novel sense and antisense transcription at the TRPM2 locus in cancer. Cell Res..

[61] Zheng J., Liu F., Du S., Li M., Wu T., Tan X., Cheng W. (2019). Mechanism for Regulation of Melanoma Cell Death via Activation of Thermo-TRPV4 and TRPV2. J. Oncol..

[62] Meierjohann S. (2014). Oxidative stress in melanocyte senescence and melanoma transformation. Eur. J. Cell Biol..

[63] Lin J.Y., Fisher D.E. (2007). Melanocyte biology and skin pigmentation. Nature.

