# Supplementary material for: The TRPA1 Channel Amplifies the Oxidative Stress Signal in Melanoma
Source: Cells. 2021 Nov 11;10(11):3131. doi: 10.3390/cells10113131 (PMC8624842; doi:10.3390/cells10113131)
Supplement: Supplementary file 1 [file cells-10-03131-s001.zip › cells-1443606-supplementary.pdf]

Supplementary Material

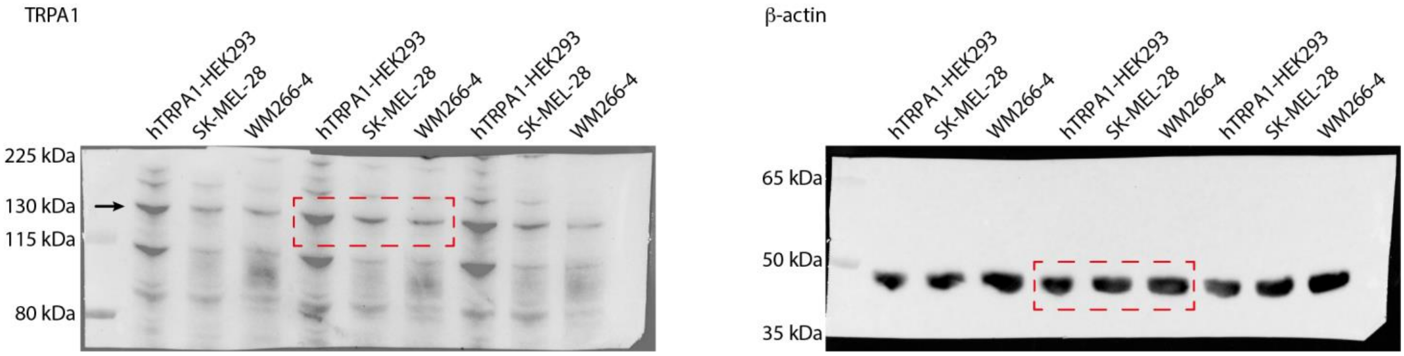

Figure S1. The uncropped scan of the blot. The blot area within dashed line box is shown in the Figure 3c.
